# Acute Paraoxon-Induced Neurotoxicity in a Mouse Survival Model: Oxidative Stress, Dopaminergic System Alterations and Memory Deficits

**DOI:** 10.3390/ijms252212248

**Published:** 2024-11-14

**Authors:** Edurne Urquizu, Selma Paratusic, Júlia Goyenechea, Cristian Gómez-Canela, Berta Fumàs, David Pubill, Demetrio Raldúa, Jordi Camarasa, Elena Escubedo, Raúl López-Arnau

**Affiliations:** 1 Department of Pharmacology, Toxicology and Therapeutic Chemistry, Pharmacology Section and Institute of Biomedicine (IBUB), Faculty of Pharmacy, University of Barcelona, 08028 Barcelona, Spain; edurneurquizullop@ub.edu (E.U.);; 2 Department of Analytical Chemistry and Applied (Chromatography Section), School of Engineering, Institut Químic de Sarrià—Universitat Ramon Llull, 08017 Barcelona, Spain; 3 Institute for Environmental Assessment and Water Research (IDAEA-CSIC), 08034 Barcelona, Spain

**Keywords:** organophosphate, paraoxon, memory, oxidative stress, mouse

## Abstract

The secondary neurotoxicity induced by severe organophosphorus (OP) poisoning, including paraoxon (POX), is associated with cognitive impairments in survivors, who, despite receiving appropriate emergency treatments, may still experience lasting neurological deficits. Thus, the present study provides a survival mouse model of acute and severe POX poisoning to examine secondary neurotoxicity. Swiss CD-1 male mice were injected with POX (4 mg/kg, s.c.) followed by atropine (4 mg/kg, i.p.), pralidoxime (2-PAM; Pyridine-2-aldoxime methochloride) (25 mg/kg, i.p., twice, 1 h apart) and diazepam (5 mg/kg, i.p.), resulting in a survival rate >90% and Racine score of 5–6. Our results demonstrated that the model showed increased lipid peroxidation, downregulation of antioxidant enzymes and astrogliosis in the mouse hippocampus (HP) and prefrontal cortex (PFC), brain areas involved in cognitive functions. Moreover, dopamine (DA) levels were reduced in the hp, but increased in the PFC. Furthermore, the survival mouse model of acute POX intoxication did not exhibit phenotypic manifestations of depression, anxiety or motor incoordination. However, our results demonstrated long-term recognition memory impairments, which are in accordance with the molecular and neurochemical effects observed. In conclusion, this mouse model can aid in researching POX exposure’s effects on memory and developing potential countermeasures against the secondary neurotoxicity induced by severe OP poisoning.

## 1. Introduction

POX is the main active and toxic metabolite of parathion, an OP compound that has been widely used as pesticide in agriculture and industry. However, it has also been used as a chemical weapon in several wars, criminal acts and terrorist attacks, leading to a serious health concern worldwide [1]. The OP compounds, including POX, irreversibly inhibit acetylcholinesterase (AChE), resulting in acute cholinergic syndrome, which can lead to seizures, loss of consciousness and even death [2,3].

The current standard emergency therapy for severe acute OP poisoning includes atropine, which prevents hyperactivation of muscarinic receptors by excessive acetylcholine (ACh) synaptic levels, oximes to reactivate AChE and benzodiazepines to control seizures and prevent death by increasing GABAergic signaling [4]. Although the existing emergency treatment is effective at partially reversing some of the effects of AChE inhibition, a cascade of downstream events occurs after POX intoxication, leading to a secondary neuronal toxicity [5,6]. Thus, survivors of acute OP poisoning suffer from significant morbidities, including deficits in cognition [7,8,9,10]. In this sense, the hippocampus (HP) and prefrontal cortex (PFC), which are involved in different mood disorders, exhibit an enhanced vulnerability to OP compounds, probably due to the high density of cholinergic innervations [11]. In fact, it is known that generation of reactive oxygen species (ROS) in such brain areas may play an important role in the mechanism behind the complications associated with severe acute OP poisoning (e.g., neuroinflammation, memory impairments, etc.) [12,13,14]. Moreover, it is known that certain neurotoxins, like POX, may have actions beyond AChE activity, affecting other neurotransmitter (NT) systems (i.e., dopaminergic, serotoninergic, GABAergic), which may be involved in the propagation and or exacerbation of the brain damage [15,16,17].

The Food and Drug Administration (FDA) has been using the Animal Efficacy Rule to approve treatments to reduce or prevent the toxicity induced by life-threatening toxic agents such as OP poisoning, since human efficacy trials are not feasible or ethical. Thus, the FDA mainly relies on animal models to provide enough evidence of treatment effectiveness [18]. Therefore, most studies of the acute and delayed toxicity induced by OP exposure have been carried out in experimental animals (rats, guinea pig and nonhuman primates) although the effectiveness of medical therapies against OP intoxication in humans is species specific and can be more reliably predicted in guinea pigs and nonhuman primates than in rodents [18]. Nevertheless, a survival rodent model of OP poisoning (using the current standard emergency therapy) would also be desirable since it can be employed to study some of the molecular bases of OP-induced chronic cognitive comorbidities, neuronal injury and develop suitable therapies. For example, preclinical rat models of OP poisoning have demonstrated persistent neuropathology and/or behavioral deficits, such as memory impairments [19,20,21,22,23,24], that mimic long-term effects observed in human survivors of acute OP intoxication [3]. Additionally, some evidence suggests that males may be more susceptible than females to a variety of adverse neurological outcomes associated with OP exposure across the lifespan (for a review see [25]).

To our knowledge, there is neither data on mid-/long-term cognitive and behavioral impairment nor oxidative stress biomarkers in a survival mouse model of severe acute OP poisoning that mimics the use of the standard emergency therapy. Such work is also important in view of the secondary neuropsychological deficits suggested by several epidemiological studies [26,27,28]. Moreover, the main benefits of a mouse model are (i) the availability of transgenic strains that could be leveraged to investigate pathogenic mechanisms underlying the neurotoxicity of acute OP poisoning; (ii) the relatively small size; and (iii) short generation time and price in comparison to rat, guinea pig, and nonhuman primate models.

Therefore, the main goal of the present study is to develop a survival mouse model of OP acute poisoning to characterize the neurotoxic effects including oxidative stress biomarkers in specific brain areas (mainly HP and PFC), NT system alterations, cognitive and neurobehavioral declines. Moreover, this model can be useful to investigate and evaluate novel therapeutics for secondary neurotoxicity derived from this severe acute poisoning.

## 2. Results

### 2.1. Preliminary Experiment: Effect of POX Treatment on Racine Scale and Survival Rate

Behavioral responses were examined after POX (2, 3, or 4 mg/kg) administration and all the corresponding antidotes. All subjects entered Status Epilepticus (S.E.) and presented observable changes such as orofacial movements, chewing, myoclonus, Straub tail reaction, bilateral convulsions of extremities and tremors. Specifically, 4 mg/kg also caused stereotypic movements, falls and complete tonic convulsions.

**Figure 1:**
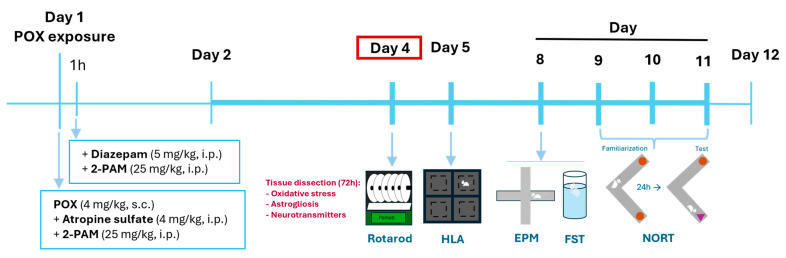
Schematic representation of the experimental procedure. Schedule of the behavioral tests performed and biochemical analysis.

Changes in behavior were observed for 60 min and were scored using a modified Racine Scale scoring from 0 to 6 (see Supplementary Materials). Observations showed that the timepoint of the beginning of S.E., the Racine Scale scores and the mortality rate changed when increasing the POX dose (see Table S1). The highest dose tested (4 mg/kg) caused an earlier onset of the S.E.; the most severe symptoms scored within the Racine Scale and a survival rate > 90% (Table S1). All animals that survived the acute POX challenge at 10 min lived to complete the 12-day experimental protocol. Therefore, the dose of 4 mg/kg of POX was chosen as the definitive treatment to perform all the behavioral and biochemical studies (Figure 1).

### 2.2. Behavioral Effects After Acute POX Administration (4 mg/kg, Subcutaneously [s.c.]) Plus Emergency Treatment

#### 2.2.1. Motor Coordination (Rotarod Test)

Unpaired student *t*-test analysis revealed no significant effect of the POX treatment neither in the Rotarod score (Figure S1A) (t_(24)_ = 0.7814, *p* > 0.05) nor in the latency to fall from the rod (Figure S1B) (t_(24)_ = 0.5964, *p* > 0.05), pointing to a lack of motor coordination impairment.

#### 2.2.2. Basal Locomotor Activity

**Figure 2:**
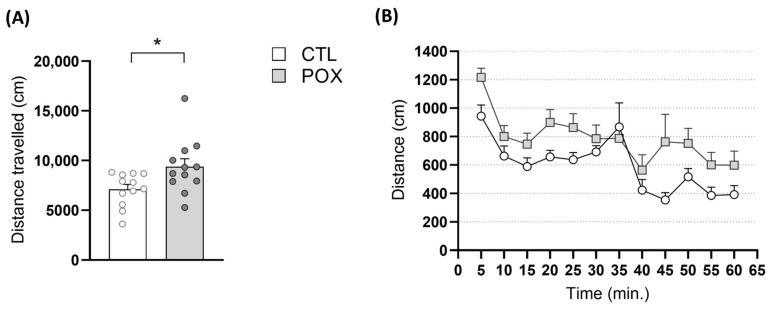
Basal locomotor activity of Swiss CD-1 male mice treated with POX plus standard emergency treatment in comparison with vehicle-treated mice (CTL). (**A**) Total travelled distance (cm) in 60 min (* *p* < 0.05, unpaired Student’s *t*-test) and (**B**) time course profile of the distance (cm) travelled in 5 min clusters for 1 h. Bars represent the mean ± Standard Error of the Mean (SEM), N = 10–13/group.

As shown in Figure 2A, unpaired Student’s *t*-test analysis of the total distance travelled revealed a significant increase in the basal locomotor activity of POX-treated animals in comparison to the control group (t_(23)_ = 2.24, *p* < 0.05).

Horizontal Locomotor Activity (HLA) time course profile is also represented in Figure 2B. Two-way ANOVA yielded a significant effect of the variables Time and Treatment, but not for the interaction between both factors (Interaction: F_(11,242)_ = 1.221, *p* > 0.05; Time: F_(11,242)_ = 10.46, *p* < 0.001; Treatment: F_(1,22)_ = 5.862, *p* < 0.05).

#### 2.2.3. Anxiety-like Effects (Elevated Plus Maze)

As shown in Figure S2, two-way ANOVA revealed a significant main effect of the variable Arm (F_(1,48)_ = 152.1, *p* < 0.001) but there was neither a significant result for the Treatment nor the Interaction (Treatment: F_(1,48)_ = 0.8974, *p* > 0.05; Interaction: F_(1,48)_ = 0.2470, *p* > 0.05).

#### 2.2.4. Depressive-like Symptoms (Forced Swim Test (FST))

As shown in Figure S3, POX treatment did not modify the animals’ immobility time in the FST in comparison with control group (t_(12)_ = 0.3273, *p* > 0.5).

#### 2.2.5. Memory Impairments (Novel Object Recognition Test (NORT))

**Figure 3:**
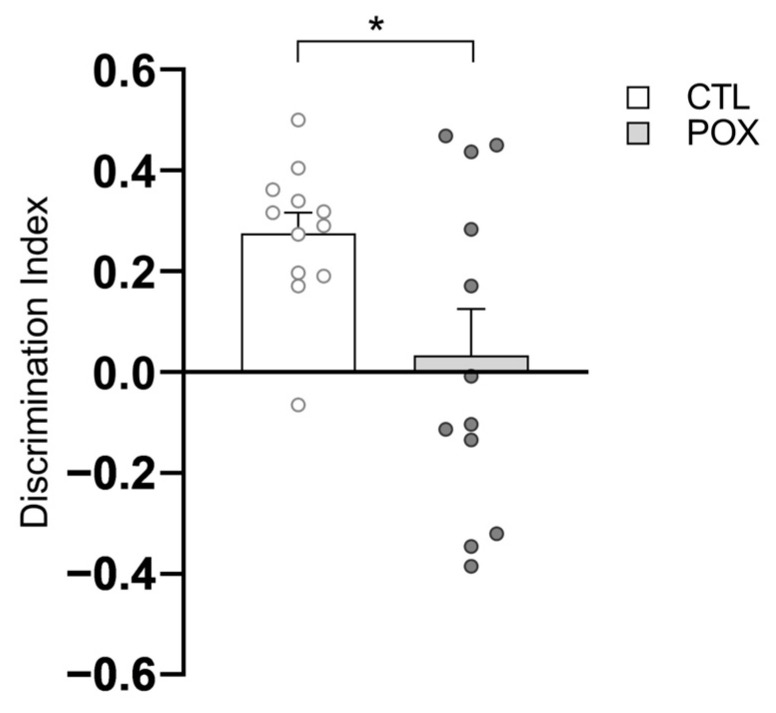
Recognition memory evaluation by NOR test in control and POX survivor mice. Rate of discrimination presented by the DI of both the familiar and novel object by the control and POX group. Data are expressed as mean ± SEM, * *p* < 0.05, unpaired Student’s *t*-test, N = 12/group.

In the testing phase of NORT, unpaired Student’s *t*-test revealed no significant differences in the total exploration time of both the familiar and novel object by control and POX group (t_(24)_ = 1.494, *p* > 0.05, see Figure S4). Notably, a significantly lower discrimination index (DI) in POX-treated mice in comparison with saline-treated animals (t_(22)_ = 2.394, *p* < 0.05) (Figure 3) was observed, suggesting a decline in recognition memory in the survival mouse model of POX acute intoxication.

### 2.3. Biomarkers of Oxidative Stress

To analyze the neurotoxicity caused by the acute administration of POX plus the emergency treatment, we analyzed the expression of several antioxidant enzymes and oxidative stress markers 72 h after POX exposure.

**Figure 4:**
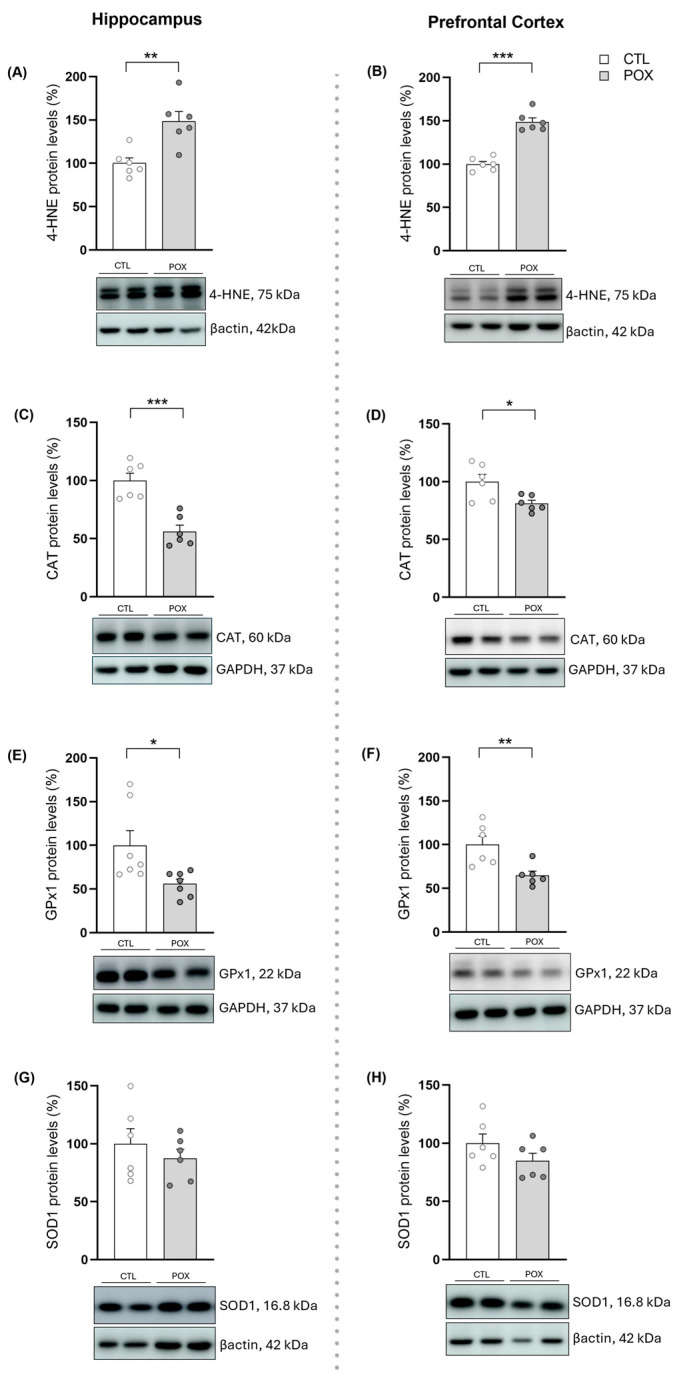
Protein levels (%) related to oxidative stress response in the HP (**A**,**C**,**E**,**G**) and PFC (**B**,**D**,**F**,**H**) after POX plus standard emergency treatment in comparison with vehicle-treated mice (CTL). The 4-HNE (**A**,**B**), CAT (**C**,**D**), GPx-1 (**E**,**F**) and SOD-1 (**G**,**H**) protein changes were determined 72 h after treatment. Data are expressed as mean ± SEM, * *p* < 0.05, ** *p* < 0.01, *** *p* < 0.001, unpaired Student’s *t*-test, N = 6–7/group. Representative blots were included below each graph. To see the uncropped original images, see Supplementary Materials.

As shown in Figure 4, the lipid peroxidation product 4-Hydroxynonenal (4-HNE) protein levels were significantly increased both in the HP (t_(10)_ = 3.773, *p* < 0.01) and the PFC (t_(10)_ = 8.764, *p* < 0.001) of POX-treated subjects (Figure 4A,B). Moreover, POX treatment significantly decreased the protein levels of the enzymes Catalase (CAT) and Glutathione Peroxidase 1 (GPx1) in the HP (t_(10)_ = 3.957, *p* < 0.01 and t_(12)_ = 2.506, *p* < 0.05, respectively) and PFC (t_(10)_ = 2.734, *p* < 0.05 and t_(10)_ = 3.266, *p* < 0.01, respectively) (Figure 4C–F). However, POX treatment did not significantly affect the protein levels of Superoxide Dismutase 1 (SOD1) 72 h after treatment (Figure 4G,H).

### 2.4. NT Analysis and Dopamine Transporter (DAT) Quantification

**Figure 5:**
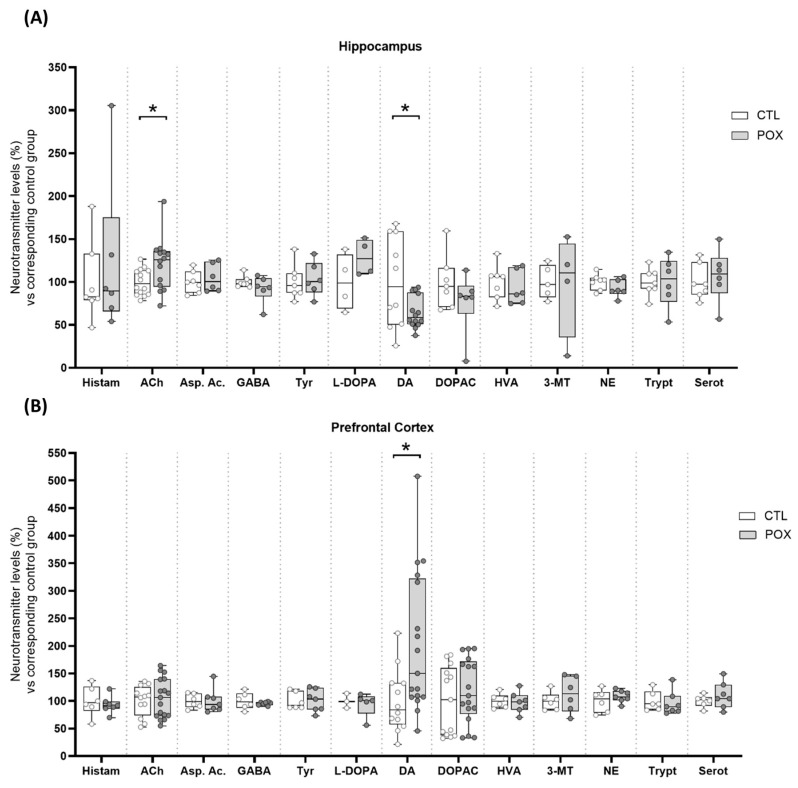
NT levels (expressed as percentage vs. corresponding control group) in the HP (**A**) and PFC (**B**). Bars represent mean ± SEM, * *p* < 0.05, Student’s *t*-test, N = 6–17/group. Histam = histamine; Asp. Ac. = aspartic acid; GABA = gamma-aminobutyric acid; Tyr = tyrosine; L-DOPA = levodopa; DOPAC = 3,4-Dihydroxyphenylacetic acid; HVA = homovanillic acid; 3-MT = 3-Methoxytyramine; NE = norepinephrine; Tryp = tryptophan; Serot = serotonin.

**Figure 6:**
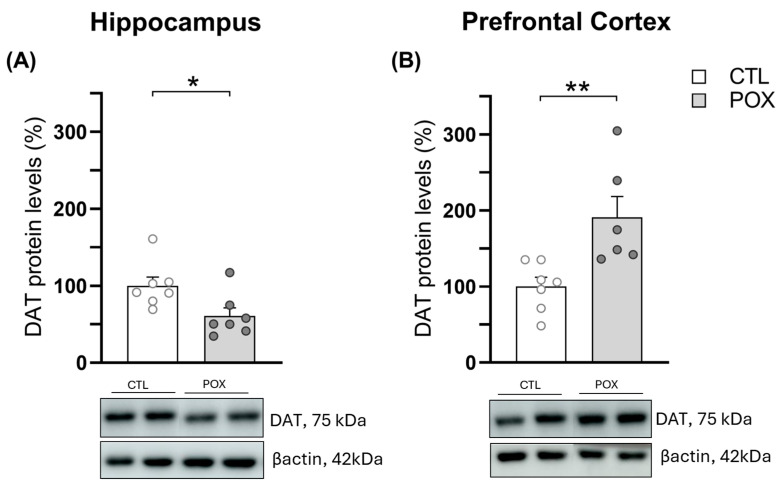
DAT protein expression (%) in HP (**A**) and PFC (**B**) 72 h after POX plus standard emergency treatment in comparison to vehicle-treated mice (CTL). Bars represent mean ± SEM, * *p* < 0.05, ** *p* < 0.01. Unpaired Student’s *t*-test, N = 6–7/group. Representative blots were included below each graph. Original uncropped images can be found in the Supplementary Materials.

Following the same schedule sampling as in oxidative stress assays, NT levels were assessed 72 h after POX administration. The ACh concentrations were significantly increased in the HP of POX-treated animals (t_(26)_ = 2.307, *p* < 0.05) (Figure 5A). However, no changes were observed in the PFC. Furthermore, DA levels were significantly decreased in the HP (t_(22)_ = 2.335, *p* < 0.05) (Figure 5A), but significantly increased in the PFC of POX-treated mice (t_(27)_ = 2.594, *p* < 0.05) (Figure 5B). Consistent with these findings, DAT protein levels were significantly decreased in hippocampal tissue (t_(12)_ = 2.550, *p* < 0.05), while overexpressed in PFC (t_(11)_ = 3.185, *p* < 0.01) (Figure 6A,B) after POX treatment. No significant changes were observed for the other NTs analyzed (Figure 5A,B), including the ones studied in striatum (STR) (Figure S5). Concentrations of each NT (pg/mg tissue) can be found in the Supplementary Materials (Table S2).

### 2.5. Hippocampal and PFC Astrogliosis

**Figure 7:**
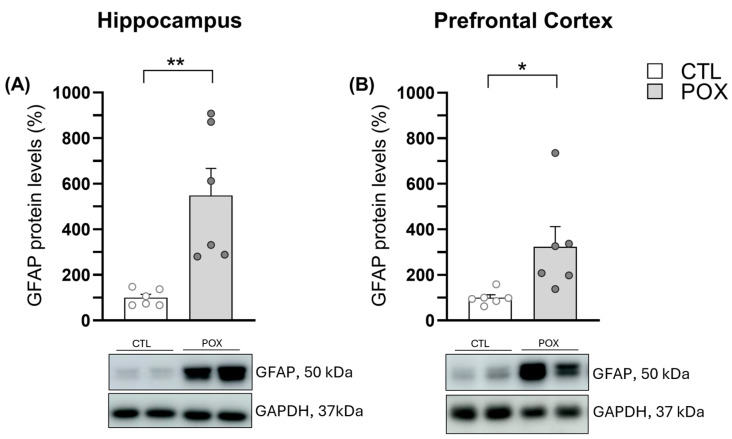
GFAP protein expression (%) in HP (**A**) and PFC (**B**) 72 h after POX plus standard emergency treatment in comparison to vehicle-treated mice (CTL). Data are expressed as mean ± SEM N= 6/group. Unpaired Student’s *t*-test, * *p* < 0.05, ** *p* < 0.01. Representative blots were included below each graph. Uncropped original images can be seen in the Supplementary Materials.

The protein expression levels of Glial Fibrillary Acidic Protein (GFAP), a major protein found in astrocytic cells of the brain and considered a marker of astrogliosis, were also assessed to investigate possible inflammatory effects caused by POX intoxication in the brain of the survivor mice. Results showed a significant increase in GFAP protein levels in both tested areas, the HP (t_(10)_ = 3.742, *p* < 0.01) (Figure 7A) and PFC (t_(10)_ = 2.512, *p* < 0.05) (Figure 7B).

## 3. Discussion

Despite the risk that many people face from the toxicity generated by OP pesticide exposure in agriculture, the threat of intentional poisoning of military and civilians with OP by a terrorist attack is also a growing concern [29]. During the last 45 years, these compounds have been used in multiple attacks such as in the Iraq–Iran War in the 1980s, the Persian Gulf War in the 1990s, terrorist attacks in Matsumoto and Tokyo, Japan in 1995 and sarin exposure on multiple occasions in Syria from 2011–2017 [29,30,31]. Given the important threat of using OPs to target military or civilians and the unfeasibility of conducting human trials, there is a need not only to understand the molecular basis and neurological consequences of OP poisoning, but also to establish in vitro and in vivo models of OP acute intoxication. In this sense, the present research provides a survival mouse model of acute and severe POX poisoning to study the secondary neurotoxicity, to improve and develop novel medical countermeasures against the morbidity and delayed neurotoxicity induced by these chemical warfare agents.

It is widely known that acute exposure to OP compounds causes Status Epilepticus (S.E.) [22]. This state of seizure activity has been linked to a secondary toxicity characterized by oxidative stress and inflammation associated with further neurological lesions and cognitive impairments in surviving rodents [22,24,32,33]. In this sense, all POX doses used in the present study, together with the corresponding standard emergency treatment (atropine, 2-PAM and diazepam), produced rapid behavioral responses and caused S.E. in mice due to an initial cholinergic crisis, similar to that observed in humans [34]. However, the highest dose tested of POX (4 mg/kg) induced the most severe symptoms according to the Racine Scale, maintaining a survival rate higher than 90%, which points out neuronal damage and allows the performance of further studies with such subjects.

During this cholinergic overstimulation, many molecular events occur, leading to generation of free radicals and reactive oxygen species (ROS) which may disrupt macromolecules and damage proteins, lipids and nucleic acids. Particularly, ROS can react with lipid membranes and cause lipid peroxidation, producing reactive secondary products such as 4-HNE, recognized as trigger for oxidative stress [35,36,37,38,39]. In this regard, the survival mouse model of POX acute intoxication used in the present study showed increased 4-HNE protein levels in both HP and PFC brain areas, corroborating the impairment of cellular homeostasis and disruption of lipidic membranes previously observed in rodents following acute and chronic OP exposure [38,40,41].

In normal metabolism, antioxidant defenses convert ROS into less toxic molecules, protecting the tissues from damage, and antioxidant enzymes such as SOD1, CAT and GPx1. These defenses play an important role in preventing such oxidative injury. In the present work, CAT and GPx1 appear to be downregulated in the tested mouse brain areas (HP and PFC) 72 h after POX exposure, probably as a consequence of increased levels of ROS [42]. In fact, Jafari and colleagues (2012) reported a decrease in CAT enzyme activity in whole brain homogenate at the dose of 1.5 mg/kg of POX without administering any emergency treatment [40]. Moreover, chronic treatment with other OP compounds, such as malathion and diisopropyl phosphoro-fluoridate (DFP), have also been shown to decrease CAT and GPx1 levels and/or enzyme activity in whole brain homogenates in treated mice [38,41]. Altogether, our results demonstrate that a single dose of 4 mg/kg of POX plus the standard emergency treatment still generates radicals that damage lipidic membranes and disrupts antioxidant defenses, leading to a state of oxidative stress in mice [42,43]. In fact, this state has already been reported in several studies focused on OP intoxication in humans [14,44,45] and rodents [40,41,46].

Oxidative stress and neuroinflammation after OP exposure are two connected processes, as this imbalance between ROS and antioxidant enzymes may trigger changes in redox homeostasis and neuroinflammation [23,47], which are implicated in the pathogenesis of various neurological disorders. In fact, previous studies have already demonstrated that exposure to an acute dose of OP is enough to cause an inflammatory response. The study carried out by Zare and colleagues (2020) demonstrated an increased number of GFAP-positive cells in rat PFC after treatment with a convulsive dose (0.7 mg/kg or 1 mg/kg) of POX [48]. Moreover, an acute exposure to DFP or soman in rats also evidenced that astrogliosis is a strong neuronal response within days after suffering S.E. [49]. Furthermore, Maupu and co-authors observed an increase in GFAP levels in the HP after an acute DFP administration to mice [33]. The present study demonstrates that, although the standard emergency treatment could increase survival rate [24] and ameliorate the acute cholinergic syndrome induced by POX administration, survivor mice still possess increased protein levels of GFAP 72 h after POX exposure in both brain areas tested, HP and PFC. In fact, because inflammatory markers appear gradually over time, some studies have suggested that inflammation is likely to contribute to neuronal damage in the mid-to-long term rather than in the immediate lethality caused by the OP compound [50]. Moreover, it must be pointed out that the astrogliosis observed in the present survival mouse model of POX intoxication runs in parallel with the dysregulation of oxidative stress markers discussed above, upholding evidence of a link between both processes.

It is known that OP compounds irreversibly inhibit AChE, which leads to an increase in ACh in the synaptic cleft causing the cholinergic syndrome. In this sense, the NT analysis performed in the current study revealed that total ACh levels remained elevated 72 h post-POX exposure in the HP of POX-surviving mice, pointing to a widespread disruption of cholinergic signaling beyond just synaptic clefts. This broader disruption may contribute to persistence and/or exacerbation of neuronal damage in this brain region.

As mentioned previously, civilians and soldiers who underwent acute or repeated OP exposure presented mid-/long-term neurologic and psychiatric impairments. Among these deficiencies, there were attention and learning deficits, memory loss, depression-like behavior, anxiety and psychotic episodes, as well as motor dysfunctions [18,29]. In the current study, the survival mouse model of acute POX intoxication did not exhibit phenotypic manifestations of depression, anxiety or motor coordination deficits. However, our results demonstrated long-term recognition memory impairments assessed by NORT. Given the well-established role of HP in memory and learning [51,52], it is noteworthy that such brain area of POX-surviving animals showed a pronounced state of oxidative stress and astrogliosis, which could contribute to the memory deficits observed [24,32]. Furthermore, the HP is modulated by cholinergic and DAergic systems, among others, and both NTs are crucial for coordinating hippocampal functions like memory and learning [53,54]. Particularly, some studies focused on the relationship between DA and hippocampal function have demonstrated that low levels of DA or unbalanced DAergic system can significantly affect hippocampal activity and are associated with cognitive deficits and altered plasticity [55,56]. In this sense, our results revealed a decrease in DA concentration and a significant reduction in DAT expression in the HP of POX-surviving mice. These findings suggest a potential disruption in DAergic signaling within the HP following POX-exposure, which could be an important factor underlying the memory deficits observed. Although the effects of POX on the DAergic system in hippocampal tissue remain not fully explored, a previous study also demonstrated a decrease in hippocampal DA levels following sublethal doses of O-Ethyl-S-[2(diisopropylamino)ethyl] methylphosphonothioate (also known as VX), another OP compound, in mice [57].

On the other hand, the PFC also plays a pivotal role in cognitive functions, including memory and decision-making [58]. Parallel to what was seen in HP, our results demonstrated that the PFC of POX-surviving mice also exhibited disruptions in oxidative stress biomarkers as well as astrogliosis, both of which may also contribute to and/or exacerbate the observed memory impairment [59,60,61]. However, in contrast to the NT analysis and DAT density results in the HP, DA concentration and DAT protein levels were elevated in the PFC of survivor animals following POX administration. This finding is in line with other studies demonstrating that excessive DAergic system activation in PFC is linked to impaired cognitive performance, including memory and attention [62,63] (for a review see [64]). Moreover, Oswal and colleagues (2013) also demonstrated that low-dose sarin exposure, another OP nerve agent capable of producing memory impairments [65,66], induced long-term increases in DA levels in mouse PFC [67].

Prior studies have reported that young animals exposed to OP compounds can show changes in locomotor activity depending on age, OP compound, timepoint, species or strain used, among other variables [16,68,69,70,71,72,73,74,75]. In this sense, our results demonstrated that Swiss CD-1 male mice treated with POX (4 mg/kg, intraperitoneally [i.p.]) and the standard emergency treatment exhibited a significant increase in basal locomotor activity 5 days after POX administration. It is known that DA regulates locomotion activities, as procedures that decrease DA activity in neural systems lead to hypoactivity, and several drugs that increase DA transmission cause hyperlocomotion [76], particularly in STR [77]. Moreover, some studies have also shown that POX exposure can acutely trigger increased DA release in STR [78,79]. Therefore, we decided to analyze any changes in NT levels in this brain area. However, no changes in DA content were observed in the STR. Thus, further studies are needed to elucidate the exact mechanism responsible for such observed hyperlocomotion.

In summary, the survival mouse model of acute POX intoxication developed in the present study showed long-term memory impairments that were correlated with dysregulation of oxidative stress markers, astrogliosis, and disruption of DAergic neurotransmission in brain regions involved in memory and cognition, such as the HP and PFC. Additionally, the survivors also exhibited an increase in basal locomotor activity. Finally, our results highlight the utility of this model to study the molecular, neurochemical and behavioral consequences of acute OP exposure, especially those related to memory impairments, and to subsequently develop potential treatments to mitigate the chronic morbidities associated with OP poisoning.

## 4. Materials and Methods

### 4.1. Animals

Male (7–8 weeks-old) Swiss CD-1 mice (Janvier, Le Genest-Saint-Isle, France) weighing 30–40 g were used. Animals were housed 6 to 7 per cage and housing conditions were the same for all mice, with controlled temperature (22 ± 1 °C) and with a cycle of 12 h light/dark with availability of standard food and water at any time. Animal care and experiments were carried out under the guidelines of the European Community Council (2010/62/EU) and were approved by the Animal Ethics Committee of the University of Barcelona under the supervision of the Autonomous Government of Catalonia. This study fulfills the ARRIVE guidelines for experiments involving animals.

### 4.2. Drugs and Materials

Paraoxon-ethyl (ref. 04-C15850000) was purchased from Cymit quimica (Barcelona, Spain) and was dissolved with cold Phosphate Buffered Saline 0.01 M. Atropine sulfate (ref. A0257-5G) and 2-PAM (ref. P9053-5G) purchased from Sigma Aldrich (St. Louis, MO, USA) and prepared in 0.9% NaCl. Diazepam (ref. D0899-100MG) was obtained from Sigma Aldrich (St. Louis, MO, USA) and was dissolved in saline solution with 2% DMSO (ref. SU01511000, Scharlab; Barcelona, Spain) and 5% Kolliphor (ref. 42966; Sigma, St. Louis, MO, USA). All reagents were prepared immediately before administration. The protease inhibitor cocktail (ref. 539131) and phosphatase inhibitor (sodium orthovanadate; ref. 450243) were acquired from Sigma Aldrich (St. Louis, MO, USA). All the other reagents were analytical grade and were obtained from different commercial sources. Crystalline solid standards of DA hydrochloride (ref. H8502), DOPAC (ref. 850217), and ACh (ref. A6625) were purchased by Sigma Aldrich (St. Louis, MO, USA). Isotopically labeled standards, such as DA-1,1,2,2-d4 (ref. TRC-D533782-1MG) and DOPAC-d5 (TRC-D454253) were purchased from Toronto Research Chemicals (Toronto, ON, Canada). Stock standard solutions of both unlabeled and labelled neurochemicals, were prepared at 1 µg µL^−1^ in MeOH or ultra-pure water and kept at −20 °C in the dark (in silanized amber vial). However, standard solutions were prepared using 90:10 Acetonitrile (ACN)/ultra-pure water + 1% Formic Acid (FA) mixture as solvent. Internal standard mixture (ISM), containing all labeled standards, was prepared in the same solvent, and was used as the internal standard for the entire analytical process. The ACN (LC–MS grade, ref. 1.00029.2500) was purchased from VWR Chemicals (Leuven, Belgium). The FA (ref. 10780320) was acquired from Fischer Scientific (Loughborough, UK). Ammonium formate (NH4COOH) (ref. 70221) was bought from Sigma Aldrich (St. Louis, MO, USA). Ultra-pure water was obtained daily through the Millipore Milli-Q purification system (Millipore, Bedford, MA, USA).

### 4.3. In Vivo Treatment

Mice were injected s.c. with an acute dose of 4 mg/kg of POX, followed 1 min later by an i.p. administration of atropine (4 mg/kg) and 2-PAM (25 mg/kg), thus increasing the survival rate of the treated subjects (the dose of POX was chosen according to preliminary experiments; see Section 2.1 in Results). Within 3 to 5 min of POX administration, mice entered an S.E. that was monitored for 1 h using a modified Racine Scale [80,81] with scores ranging from 0 to 6 (see Supplementary Materials). One hour after POX exposure, mice were administered i.p. with 2-PAM (25 mg/kg) and diazepam (5 mg/kg) to control and terminate seizures. The dose of diazepam was chosen according to previous studies [82]. At the end of the treatment, all subjects were administered 0.9% NaCl s.c. to counteract possible dehydration from all the cholinergic symptoms. Control mice underwent the same treatment, but were injected with all vehicles without the corresponding drugs. All mice were closely supervised until the day of their euthanasia, by monitoring their weight, appearance and behavior.

### 4.4. Behavioral Assays

#### 4.4.1. Rotarod

To evaluate possible motor impairments, subjects underwent the rotarod test on day 4 after treatment. For this experiment, a Panlab RotaRod (Harvard Apparatus) apparatus with the capability to test up to 5 subjects simultaneously was used. Subjects were placed into the lanes of the rotor at an initial speed of 4 revolutions per minute (rpm) and with a continuous acceleration rate of 20 revolutions/min^2^. The rod of the apparatus accelerated up to a maximum velocity of 40 rpm, which was maintained over time. Mice falling from the rod landed into the lever of the corresponding lane, thus stopping the timer that reported the time spent on the rod and the speed at which the subject fell [83]. The procedure was repeated for a total of 3 sessions per subject, with a separation of 2 h. The latency time and the score of the speed were used as parameters for analysis.

#### 4.4.2. HLA

Five days after POX administration, mice were placed into a black Plexiglas arena (25 × 25 × 40 cm) in a behavioral testing room under diminished light intensity and white noise. Their basal locomotor activity was recorded by a digital camera and tracked by a specific software program (Smart v3.0, Panlab, Barcelona, Spain) for 1 h. Four subjects were recorded simultaneously as the apparatus is divided into 4 identical arenas. The total distance travelled by each subject was analyzed [84].

#### 4.4.3. Elevated Plus Maze (EPM)

This test is used to assess anxiety-related behavior in mice and is based on the aversion of rodents for open bright spaces. The maze is made of grey PVC and consists of 4 arms (32 cm long × 6.5 cm wide) connected by a square center and elevated 43 cm from the floor. Two of the arms are surrounded by 14 cm walls and are placed opposite the other 2 arms, which are open without walls [85]. On day 8 after acute POX exposure, the maze was placed in a behavioral testing room with dim light intensity (30 lux). Video-tracking software (Smart v3.0, Panlab, Barcelona, Spain) was used to record and collect data automatically as the subjects were allowed to freely explore the maze. The procedure consists of placing the individuals in the center of the maze facing the open arm and recording their behavior for 5 min. At the end of each session, subjects were removed from the maze, and it was cleaned with 70% ethanol, removing any scent from the previous individuals. The analysis was made by calculating the number of entries and preference for each arm to measure anxiety-like behavior, which is directly correlated with the predisposition for spending more time in closed arms.

#### 4.4.4. FST

This behavioral assay was performed to assess potential despair-like states in the treated mice. Eight days after acute POX exposure, mice were placed in a glass cylindrical tank (24 cm high × 11.5 cm diameter) filled up with water set at a stable temperature (23–25 °C). Their behavior was recorded with a digital camera for 6 min and at the end of each session, mice were taken out of the tank and dried with a towel. Active and passive behavior was evaluated by analyzing the last 4 min of the recording, thus indicating the state of the animal. Active behavior is defined as climbing, swimming and making quick movements. Immobility time is understood as the absence of any movement except for those necessary for floating and keeping the nose above the water [86]

#### 4.4.5. NORT

In this test, mice were placed into a V-shaped [87] grey plexiglass maze of 37 × 20 × 8 cm in a behavioral testing room with attenuated and controlled light intensity (30–50 lux). The test consisted of 3 sessions separated by 24 h, which were carried out on days 9, 10 and 11 after treatment, to assess long-term recognition memory. Mice were habituated individually in the first session, allowing them to freely explore the arena for 9 min. On the second day, two identical objects were placed in the extremities of the maze leaning into the wall and subjects were able to explore them for 9 min while being recorded with a digital camera. On the day of the test (third day), one of the familiar objects was replaced by a novel object, and subjects were placed in the maze to explore them for 9 min while being recorded. Objects varied in shape, color and texture and were previously validated to guarantee no preference. The maze and the objects were cleaned between individuals with 70% ethanol to eliminate any scent from other subjects. The DI was determined by the difference in the time of exploration of the novel and familiar object divided by the total time of exploration and was used for evaluating preferences. Low preference for the novel object (lower DI) is widely linked to an impairment in recognition memory [87].

### 4.5. Bio- and Neurochemical Assays

#### 4.5.1. Tissue Sample Preparation and Protein Extraction

Mice were euthanized by cervical dislocation 72 h after POX treatment to extract protein for performing Western Blotting. The HP, STR and PFC were quickly dissected out and kept at −80 °C until use. Tissue samples were homogenized at 4 °C in 20 volumes of Tris–HCl lysis buffer with protease inhibitor cocktail and phosphatase inhibitors. The homogenate samples were vortexed for 15 s and placed in an orbital shaker for 2 h at 4 °C with a shake and roll program. Samples were afterwards centrifuged at 15,000× *g* for 30 min at 4 °C and supernatants were collected and kept at −80 °C until use. Protein concentration was measured by using the Bio-Rad Protein Reagent (ref. #5000006; BioRad, Hercules, CA, USA).

#### 4.5.2. Western Blotting and Immunodetection

A general Western blotting and immunodetection protocol was followed to analyze the expression of antioxidant enzymes such as SOD1, GPx1 and CAT, as well as the lipid peroxidation final product 4-HNE. Moreover, the protein GFAP was also studied through this technique and the expression of the DAT. For each sample, 10–15 μg of protein was mixed with sample buffer (0.5 M Tris–HCl, pH = 6.8, 10% glycerol, 2% (*w*/*v*) SDS, 5% (*v*/*v*) 2-β-mercaptoethanol, 0.05% bromophenol blue), boiled 5 min at 95 °C and loaded onto 10–12% acrylamide gels. Gels were electrophoresed and immediately transferred to Polyvinylidene Fluoride (PVDF) membranes (Immobilon-P; Merck, Darmstadt, Germany). Membranes were blocked 1 h at room temperature with WestVision Block and Diluent for Western Blots (Vector Laboratories, Newark, NJ, USA) and incubated overnight at 4 °C with a primary rabbit polyclonal to 4-HNE (ab46545, Abcam [Cambridge, UK]; dil. 1:1000); rabbit monoclonal to GPx1 (ab108427, Abcam; dil. 1:1000); sheep polyclonal anti-SOD Cu/Zn (#574597, Calbiochem [San Diego, CA, USA]; dil. 1:5000 ); rabbit monoclonal antibody to CAT (#14097, Cell Signaling [Danvers, MA, USA]; dil. 1:1000); rabbit polyclonal anti-GFAP (Z0334, Dako Omnis [Santa Clara, CA, USA]; dil. 1:5000); and mouse monoclonal anti-DAT (NBP2-22164, Novus Biological [Centennial, CO, USA]; dil. 1:1000). After washing with Tris-buffer plus 0,1% Tween-20, membranes were incubated 1 h at room temperature with their respective horse-radish peroxidase-conjugated anti-IgG antibody as follows: goat anti-mouse IgG (ab205719, Abcam; dil. 1:10,000); goat anti-rabbit IgG (ab6721, Abcam; dil. dil. 1:10,000); and rabbit anti-sheep (ab6747, Abcam; dil. 1:15,000). To visualize immunoreactive proteins, a chemiluminescence-based detection kit (Immobilon Western, Millipore, Burlington, MA, USA) and a BioRad ChemiDoc XRS gel documentation system (BioRad, Inc., Hercules, CA, USA) were used following the manufacturer’s instructions. Band densities in scanned blots were analyzed using a Bio-Rad Image Lab Software (Image Lab Software for PC Version 6.1) and protein expression levels were presented as percentage compared to control samples. As loading controls, monoclonal anti β-Actin antibody (A5441, Merck; dil. 1:2500) or monoclonal anti-GAPDH (MAB374, Sigma Aldrich; dil. 1:5000) were used, which normalized differences in protein expression.

#### 4.5.3. Neurotransmitter Extraction and Analysis

The extraction procedure of the target NTs was adapted from a previous study about NTs characterization in mouse brain [88]. Three brain areas (HP, PFC and STR) were analyzed. First, 300 µL of a cold extractant solvent (ACN:H_2_O 90:10 + 1% FA) were added to the samples, which were placed in Eppendorf tubes. All tested samples were spiked with ISM. Then, 3 stainless steel beads (3 mm diameter) were introduced in each sample. Eppendorf tubes were placed in a bead mill (TissueLyser LT, Quiagen, Hilden, Germany) to homogenize them, at 50 osc/min for 90 s. After, samples were centrifuged at 13,000 rpm for 20 min at 4 °C. The supernatant was filtered using 0.22 µM nylon filters (Scharlab, Barcelona, Spain) and kept in chromatographic vials at −80 °C until the LC–MS/MS analysis.

To analyze NTs, an Exion LCTM liquid chromatograph coupled to a triple quadrupole mass spectrometer (Triple Quad 7500 System-QTrap^®^ Ready, SCIEX, Framingham, MA, USA) equipped with an electrospray ionization source (ESI) was used. An Acquity UPLC BEH Amide (150 mm × 2.1 mm, 1.7 µm) and an Acquity UPLC BEH Amide pre-column (5 mm × 2.1 mm, 1.7 µm) (Waters, Milford, MA, USA) were used. For solvent A, a mixture of Milli-Q water and ACN (H_2_O:ACN; 95:5) containing 100 mM ammonium formate was used, and Milli-Q water and ACN (H_2_O:ACN; 15:85) containing 30 mM ammonium formate was used as solvent B. The pH was adjusted to 3 in both solvents by using FA (SensionTM + PH3, HACH^®^, Loveland, CO, USA).

In regards with the MS/MS method optimization, individual standard solutions of 1000 ng/mL were used for each of the compounds of interest. First, the wide mass-range SCAN method was performed to optimize the parent ion mass. Once this was obtained, Product Ion (PI) methods were created for each analyte using the optimal parent ion mass. These methods contained 5 experiments, each analyzed at the same m/z range applying different Collision Energies (CEs) to the precursor ion specified. Once the chromatograms were obtained, the CEs that gave the two most intense fragmentation peaks were selected to optimize two transitions for each compound (precursor ion). To complete the CE optimization, one PI method was created for each fragmentation, studying two CE values above and below the selected ones, for example, 6, 8, 10, 12 and 14 V. Next, the Q0D values for each transition of the compounds were optimized choosing the value that gave the highest intensity. Finally, the Spray Voltage (SV) of the analytes was optimized studying a range from 1500 to 5500 V, the value that gave better shape and more intense peaks was chosen. Samples with NT levels below the limit of quantification (LOQ) were excluded from the study.

### 4.6. Data Analysis

Data were expressed as mean ± SEM. Differences between groups were analyzed using unpaired Student’s *t* test and one-way ANOVA or two-way ANOVA of repeated measures followed by Tukey post-hoc test, when data were normally distributed. The alpha was set at 0.05. The statistical analysis was calculated with GraphPad Prism (version 8.0) software. Outliers were excluded following ROUT’s method (Q = 0.1%).

## Data Availability

The raw data supporting the conclusions of this article will be made available by the authors on request.

## Supplementary Materials

The following supporting information can be downloaded at: https://www.mdpi.com/article/10.3390/ijms252212248/s1. References [80,81] are cited in the Supplementary Materials.

## References

[1] Gupta R.C. (2020). Handbook of Toxicology of Chemical Warfare Agents.

[2] Figueiredo T.H., Apland J.P., Braga M.F.M., Marini A.M. (2018). Acute and Long-Term Consequences of Exposure to Organophosphate Nerve Agents in Humans. Epilepsia.

[3] Jett D.A., Spriggs S.M. (2020). Translational Research on Chemical Nerve Agents. Neurobiol. Dis..

[4] Eddleston M., Buckley N.A., Eyer P., Dawson A.H., Straub W. (2008). Management of Acute Organophosphorus Pesticide Poisoning. Lancet.

[5] McDonough J.H., McMonagle J., Copeland T., Zoeffel D., Shih T.-M. (1999). Comparative Evaluation of Benzodiazepines for Control of Soman-Induced Seizures. Arch. Toxicol..

[6] Shih T.-M., McDonough J.H. (2000). Efficacy of Biperiden and Atropine as Anticonvulsant Treatment for Organophosphorus Nerve Agent Intoxication. Arch. Toxicol..

[7] Steenland K., Jenkins B., Ames R.G., Chrislip D., Russo J. (1994). Chronic Neurological Sequelae to Organophosphate Pesticide Poisoning. Am. J. Public Health.

[8] Garcia S.J., Abu-Qare A.W., Meeker-O’Connell W.A., Borton A.J., Abou-Donia M.B. (2003). Methyl Parathion: A Review of Health Effects. J. Toxicol. Environ. Health B Crit. Rev..

[9] Johnson N.D., Duysen E.G., Lockridge O. (2009). Intrathecal Delivery of Fluorescent Labeled Butyrylcholinesterase to the Brains of Butyrylcholinesterase Knock-out Mice: Visualization and Quantification of Enzyme Distribution in the Brain. Neurotoxicology.

[10] Terry A.V., Beck W.D., Warner S., Vandenhuerk L., Callahan P.M. (2012). Chronic Impairments in Spatial Learning and Memory in Rats Previously Exposed to Chlorpyrfos or Diisopropylfluorophosphate. Neurotoxicol. Teratol..

[11] Harrison P.K., Sheridan R.D., Green A.C., Scott I.R., Tattersall J.E.H. (2004). A Guinea Pig Hippocampal Slice Model of Organophosphate-Induced Seizure Activity. J. Pharmacol. Exp. Ther..

[12] Farizatto K.L.G., McEwan S.A., Naidoo V., Nikas S.P., Shukla V.G., Almeida M.F., Byrd A., Romine H., Karanian D.A., Makriyannis A. (2017). Inhibitor of Endocannabinoid Deactivation Protects Against In Vitro and In Vivo Neurotoxic Effects of Paraoxon. J. Mol. Neurosci..

[13] Eisenkraft A., Falk A., Finkelstein A. (2013). The Role of Glutamate and the Immune System in Organophosphate-Induced CNS Damage. Neurotox. Res..

[14] Rambabu L., Megson I.L., Eddleston M. (2020). Does Oxidative Stress Contribute to Toxicity in Acute Organophosphorus Poisoning?—A Systematic Review of the Evidence. Clin. Toxicol..

[15] Raveh L., Brandeis R., Gilat E., Cohen G., Alkalay D., Rabinovitz I., Sonego H., Weissman B.A. (2003). Anticholinergic and Antiglutamatergic Agents Protect against Soman-Induced Brain Damage and Cognitive Dysfunction. Toxicol. Sci..

[16] Aldridge J.E., Meyer A., Seidler F.J., Slotkin T.A. (2005). Alterations in Central Nervous System Serotonergic and Dopaminergic Synaptic Activity in Adulthood after Prenatal or Neonatal Chlorpyrifos Exposure. Environ. Health Perspect..

[17] Mohammadi M., Ghani E., Ghasemi A., Khoshbaten A., Asgari A. (2008). Synaptosomal GABA Uptake Decreases in Paraoxon-Treated Rat Brain. Toxicology.

[18] Pereira E.F.R., Aracava Y., DeTolla L.J., Beecham E.J., Basinger G.W., Wakayama E.J., Albuquerque E.X. (2014). Animal Models That Best Reproduce the Clinical Manifestations of Human Intoxication with Organophosphorus Compounds. J. Pharmacol. Exp. Ther..

[19] Deshpande L.S., Carter D.S., Blair R.E., DeLorenzo R.J. (2010). Development of a Prolonged Calcium Plateau in Hippocampal Neurons in Rats Surviving Status Epilepticus Induced by the Organophosphate Diisopropylfluorophosphate. Toxicol. Sci..

[20] Flannery B.M., Bruun D.A., Rowland D.J., Banks C.N., Austin A.T., Kukis D.L., Li Y., Ford B.D., Tancredi D.J., Silverman J.L. (2016). Persistent Neuroinflammation and Cognitive Impairment in a Rat Model of Acute Diisopropylfluorophosphate Intoxication. J. Neuroinflamm..

[21] Hobson B.A., Rowland D.J., Sisó S., Guignet M.A., Harmany Z.T., Bandara S.B., Saito N., Harvey D.J., Bruun D.A., Garbow J.R. (2019). TSPO PET Using [^18^F]PBR111 Reveals Persistent Neuroinflammation Following Acute Diisopropylfluorophosphate Intoxication in the Rat. Toxicol. Sci..

[22] Pouliot W., Bealer S.L., Roach B., Dudek F.E. (2016). A Rodent Model of Human Organophosphate Exposure Producing Status Epilepticus and Neuropathology. Neurotoxicology.

[23] Guignet M., Dhakal K., Flannery B.M., Hobson B.A., Zolkowska D., Dhir A., Bruun D.A., Li S., Wahab A., Harvey D.J. (2020). Persistent Behavior Deficits, Neuroinflammation, and Oxidative Stress in a Rat Model of Acute Organophosphate Intoxication. Neurobiol. Dis..

[24] Deshpande L.S., Phillips K., Huang B., DeLorenzo R.J. (2014). Chronic Behavioral and Cognitive Deficits in a Rat Survival Model of Paraoxon Toxicity. Neurotoxicology.

[25] Comfort N., Re D.B. (2017). Sex-Specific Neurotoxic Effects of Organophosphate Pesticides Across the Life Course. Curr. Environ. Health Rep..

[26] Brown M.A., Brix K.A. (1998). Review of Health Consequences from High-, Intermediate- and Low-Level Exposure to Organophosphorus Nerve Agents. J. Appl. Toxicol..

[27] Jamal G.A. (1997). Neurological Syndromes of Organophosphorus Compounds. Advers. Drug React. Toxicol. Rev..

[28] Jamal G.A., Hansen S., Julu P.O.O. (2002). Low Level Exposures to Organophosphorus Esters May Cause Neurotoxicity. Toxicology.

[29] Naughton S.X., Terry A.V. (2018). Neurotoxicity in Acute and Repeated Organophosphate Exposure. Toxicology.

[30] Deluca M.A., Chai P.R., Goralnick E., Erickson T.B. (2021). Five Decades of Global Chemical Terror Attacks: Data Analysis to Inform Training and Preparedness. Disaster Med. Public Health Prep..

[31] Balali-Mood M., Abdollahi M. (2014). Basic and Clinical Toxicology of Organophosphorus Compounds.

[32] Deshpande L.S., Carter D.S., Phillips K.F., Blair R.E., DeLorenzo R.J. (2014). Development of Status Epilepticus, Sustained Calcium Elevations and Neuronal Injury in a Rat Survival Model of Lethal Paraoxon Intoxication. Neurotoxicology.

[33] Maupu C., Enderlin J., Igert A., Oger M., Auvin S., Hassan-Abdi R., Soussi-Yanicostas N., Brazzolotto X., Nachon F., Dal Bo G. (2021). Diisopropylfluorophosphate-Induced Status Epilepticus Drives Complex Glial Cell Phenotypes in Adult Male Mice. Neurobiol. Dis..

[34] Lotti M., Krieger R. (2010). Clinical Toxicology of Anticholinesterase Agents in Humans. Hayes’ Handbook of Pesticide Toxicology.

[35] Li Y., Zhao T., Li J., Xia M., Li Y., Wang X., Liu C., Zheng T., Chen R., Kan D. (2022). Oxidative Stress and 4-Hydroxy-2-Nonenal (4-HNE): Implications in the Pathogenesis and Treatment of Aging-Related Diseases. J. Immunol. Res..

[36] Pena-Llopis S. (2005). Antioxidants as Potentially Safe Antidotes for Organophosphorus Poisoning. Curr. Enzym. Inhib..

[37] Shilpa Bisht R.D. (2017). Oxidative Stress-Major Executioner in Disease Pathology. Front. Biosci..

[38] John J.J., Nagar D.P., Gujar N.L., Bhattacharya R. (2019). Oxidative and Histopathological Alterations after Sub-Acute Exposure of Diisopropyl Phosphorofluoridate in Mice: Beneficial Effect of N acetylcysteine. Life Sci..

[39] Cornelius N., Wardman J.H., Hargreaves I.P., Neergheen V., Bie A.S., Tümer Z., Nielsen J.E., Nielsen T.T. (2017). Evidence of Oxidative Stress and Mitochondrial Dysfunction in Spinocerebellar Ataxia Type 2 (SCA2) Patient Fibroblasts: Effect of Coenzyme Q10 Supplementation on These Parameters. Mitochondrion.

[40] Jafari M., Salehi M., Asgari A., Ahmadi S., Abbasnezhad M., Hajihoosani R., Hajigholamali M. (2012). Effects of Paraoxon on Serum Biochemical Parameters and Oxidative Stress Induction in Various Tissues of Wistar and Norway Rats. Environ. Toxicol. Pharmacol..

[41] Ouardi F.Z., Anarghou H., Malqui H., Ouasmi N., Chigr M., Najimi M., Chigr F. (2019). Gestational and Lactational Exposure to Malathion Affects Antioxidant Status and Neurobehavior in Mice Pups and Offspring. J. Mol. Neurosci..

[42] Matés J.M. (2000). Effects of Antioxidant Enzymes in the Molecular Control of Reactive Oxygen Species Toxicology. Toxicology.

[43] Possamai F.P., Fortunato J.J., Feier G., Agostinho F.R., Quevedo J., Wilhelm Filho D., Dal-Pizzol F. (2007). Oxidative Stress after Acute and Sub-Chronic Malathion Intoxication in Wistar Rats. Environ. Toxicol. Pharmacol..

[44] Zepeda-Arce R., Rojas-García A.E., Benitez-Trinidad A., Herrera-Moreno J.F., Medina-Díaz I.M., Barrón-Vivanco B.S., Villegas G.P., Hernández-Ochoa I., Sólis Heredia M.D., Bernal-Hernández Y.Y. (2017). Oxidative Stress and Genetic Damage among Workers Exposed Primarily to Organophosphate and Pyrethroid Pesticides. Environ. Toxicol..

[45] Ranjbar A., Solhi H., Mashayekhi F.J., Susanabdi A., Rezaie A., Abdollahi M. (2005). Oxidative Stress in Acute Human Poisoning with Organophosphorus Insecticides; A Case Control Study. Environ. Toxicol. Pharmacol..

[46] Lukaszewicz-Hussain A. (2008). Subchronic Intoxication with Chlorfenvinphos, an Organophosphate Insecticide, Affects Rat Brain Antioxidative Enzymes and Glutathione Level. Food Chem. Toxicol..

[47] Pearson J.N., Patel M. (2016). The Role of Oxidative Stress in Organophosphate and Nerve Agent Toxicity. Ann. N. Y Acad. Sci..

[48] Zare Z., Tehrani M., Zarbakhsh S., Farzadmanesh H., Shafia S., Abedinzade M., Ghanaat A., Mohammadi M. (2020). Effects of Paraoxon Exposure on Expression of Apoptosis-Related Genes, Neuronal Survival, and Astrocyte Activation in Rat Prefrontal Cortex. Neurotox. Res..

[49] Rojas A., McCarren H.S., Wang J., Wang W., Abreu-Melon J.M., Wang S., McDonough J.H., Dingledine R. (2021). Comparison of Neuropathology in Rats Following Status Epilepticus Induced by Diisopropylfluorophosphate and Soman. Neurotoxicology.

[50] Chapman S., Kadar T., Gilat E. (2006). Seizure Duration Following Sarin Exposure Affects Neuro-Inflammatory Markers in the Rat Brain. Neurotoxicology.

[51] Anand K., Dhikav V. (2012). Hippocampus in Health and Disease: An Overview. Ann. Indian. Acad. Neurol..

[52] Neves G., Cooke S.F., Bliss T.V.P. (2008). Synaptic Plasticity, Memory and the Hippocampus: A Neural Network Approach to Causality. Nat. Rev. Neurosci..

[53] Haam J., Yakel J.L. (2017). Cholinergic Modulation of the Hippocampal Region and Memory Function. J. Neurochem..

[54] McNamara C.G., Dupret D. (2017). Two Sources of Dopamine for the Hippocampus. Trends Neurosci..

[55] Chang P.K., Yu L., Chen J.C. (2018). Dopamine D3 Receptor Blockade Rescues Hyper-Dopamine Activity-Induced Deficit in Novel Object Recognition Memory. Neuropharmacology.

[56] Yang K., Broussard J.I., Levine A.T., Jenson D., Arenkiel B.R., Dani J.A. (2017). Dopamine Receptor Activity Participates in Hippocampal Synaptic Plasticity Associated with Novel Object Recognition. Eur. J. Neurosci..

[57] Graziani S., Christin D., Daulon S., Breton P., Perrier N., Taysse L. (2014). Effects of Repeated Low-Dose Exposure of the Nerve Agent VX on Monoamine Levels in Different Brain Structures in Mice. Neurochem. Res..

[58] Friedman N.P., Robbins T.W. (2022). The Role of Prefrontal Cortex in Cognitive Control and Executive Function. Neuropsychopharmacology.

[59] Bettcher B.M., Olson K.E., Carlson N.E., McConnell B.V., Boyd T., Adame V., Solano D.A., Anton P., Markham N., Thaker A.A. (2021). Astrogliosis and Episodic Memory in Late Life: Higher GFAP Is Related to Worse Memory and White Matter Microstructure in Healthy Aging and Alzheimer’s Disease. Neurobiol. Aging.

[60] Salim S. (2017). Oxidative Stress and the Central Nervous System. J. Pharmacol. Exp..

[61] Basmadjian O.M., Occhieppo V.B., Marchese N.A., Silvero C.M.J., Becerra M.C., Baiardi G., Bregonzio C. (2021). Amphetamine Induces Oxidative Stress, Glial Activation and Transient Angiogenesis in Prefrontal Cortex via AT1-R. Front. Pharmacol..

[62] Murphy B.L., Arnsten A.F.T., David Jentsch J., Roth R.H. (1996). Dopamine and Spatial Working Memory in Rats and Monkeys: Pharmacological Reversal of Stress-Induced Impairment. J. Neurosci..

[63] Bahmani Z., Daliri M.R., Merrikhi Y., Clark K., Noudoost B. (2018). Working Memory Enhances Cortical Representations via Spatially Specific Coordination of Spike Times. Neuron.

[64] Puig M.V., Antzoulatos E.G., Miller E.K. (2014). Prefrontal Dopamine in Associative Learning and Memory. Neuroscience.

[65] Kassa J., Koupilova M., Vachek J. (2001). The Influence of Low-Level Sarin Inhalation Exposure on Spatial Memory in Rats. Pharmacol. Biochem. Behav..

[66] Angrand L., Takillah S., Malissin I., Berriche A., Cervera C., Bel R., Gerard Q., Knoertzer J., Baati R., Kononchik J.P. (2021). Persistent Brainwave Disruption and Cognitive Impairment Induced by Acute Sarin Surrogate Sub-Lethal Dose Exposure. Toxicology.

[67] Oswal D.P., Garrett T.L., Morris M., Lucot J.B. (2013). Low-Dose Sarin Exposure Produces Long Term Changes in Brain Neurochemistry of Mice. Neurochem. Res..

[68] Levin E.D., Addy N., Baruah A., Elias A., Christopher N.C., Seidler F.J., Slotkin T.A. (2002). Prenatal Chlorpyrifos Exposure in Rats Causes Persistent Behavioral Alterations. Neurotoxicol. Teratol..

[69] Ricceri L., Venerosi A., Capone F., Cometa M.F., Lorenzini P., Fortuna S., Calamandrei G. (2006). Developmental Neurotoxicity of Organophosphorous Pesticides: Fetal and Neonatal Exposure to Chlorpyrifos Alters Sex-Specific Behaviors at Adulthood in Mice. Toxicol. Sci..

[70] Mohammadzadeh L., Hosseinzadeh H., Abnous K., Razavi B.M. (2018). Neuroprotective Potential of Crocin against Malathion-Induced Motor Deficit and Neurochemical Alterations in Rats. Environ. Sci. Pollut. Res. Int..

[71] Hawkey A.B., Glazer L., Dean C., Wells C.N., Odamah K.A., Slotkin T.A., Seidler F.J., Levin E.D. (2020). Adult Exposure to Insecticides Causes Persistent Behavioral and Neurochemical Alterations in Zebrafish. Neurotoxicol. Teratol..

[72] Levin E.D., Chrysanthis E., Yacisin K., Linney E. (2003). Chlorpyrifos Exposure of Developing Zebrafish: Effects on Survival and Long-Term Effects on Response Latency and Spatial Discrimination. Neurotoxicol. Teratol..

[73] Burke R.D., Todd S.W., Lumsden E., Mullins R.J., Mamczarz J., Fawcett W.P., Gullapalli R.P., Randall W.R., Pereira E.F.R., Albuquerque E.X. (2017). Developmental Neurotoxicity of the Organophosphorus Insecticide Chlorpyrifos: From Clinical Findings to Preclinical Models and Potential Mechanisms. J. Neurochem..

[74] Levin E.D., Cauley M., Johnson J.E., Cooper E.M., Stapleton H.M., Ferguson P.L., Seidler F.J., Slotkin T.A. (2014). Prenatal Dexamethasone Augments the Neurobehavioral Teratology of Chlorpyrifos: Significance for Maternal Stress and Preterm Labor. Neurotoxicol. Teratol..

[75] Baldwin K.R., Phillips A.L., Horman B., Arambula S.E., Rebuli M.E., Stapleton H.M., Patisaul H.B. (2017). Sex Specific Placental Accumulation and Behavioral Effects of Developmental Firemaster 550 Exposure in Wistar Rats. Sci. Rep..

[76] Beninger R.J. (1983). The Role of Dopamine in Locomotor Activity and Learning. Brain Res..

[77] Ikemoto S. (2002). Ventral Striatal Anatomy of Locomotor Activity Induced by Cocaine, D-Amphetamine, Dopamine and D1/D2 Agonists. Neuroscience.

[78] Alfonso M., Durán R., Fajardo D., Justo L., Faro L.R.F. (2019). Mechanisms of Action of Paraoxon, an Organophosphorus Pesticide, on in Vivo Dopamine Release in Conscious and Freely Moving Rats. Neurochem. Int..

[79] Faro L.R.F., Fajardo D., Durán R., Alfonso M. (2018). Characterization of Acute Intrastriatal Effects of Paraoxon on in Vivo Dopaminergic Neurotransmission Using Microdialysis in Freely Moving Rats. Toxicol. Lett..

[80] Figueiredo T.H., Qashu F., Apland J.P., Aroniadou-Anderjaska V., Souza A.P., Braga M.F.M. (2011). The GluK1 (GluR5) Kainate/α-Amino-3-Hydroxy-5-Methyl-4-Isoxazolepropionic Acid Receptor Antagonist LY293558 Reduces Soman-Induced Seizures and Neuropathology. J. Pharmacol. Exp. Ther..

[81] Racine R.J. (1972). Modification of Seizure Activity by Electrical Stimulation: II. Motor Seizure. Electroencephalogr. Clin. Neurophysiol..

[82] Vito S., Austin A.T., Banks C.N., Inceoglu B., Bruun D.A., Zolkowska D., Tancredi D.J., Rogawski M.A., Hammock B.D., Lein P.J. (2014). Post-Exposure Administration of Diazepam Combined with Soluble Epoxide Hydrolase Inhibition Stops Seizures and Modulates Neuroinflammation in a Murine Model of Acute TETS Intoxication. Toxicol. Appl. Pharmacol..

[83] Deacon R.M.J. (2013). Measuring Motor Coordination in Mice. J. Vis. Exp..

[84] Duart-Castells L., Nadal-Gratacós N., Muralter M., Puster B., Berzosa X., Estrada-Tejedor R., Niello M., Bhat S., Pubill D., Camarasa J. (2021). Role of Amino Terminal Substitutions in the Pharmacological, Rewarding and Psychostimulant Profiles of Novel Synthetic Cathinones. Neuropharmacology.

[85] Komada M., Takao K., Miyakawa T. (2008). Elevated plus Maze for Mice. J. Vis. Exp..

[86] Can A., Dao D.T., Arad M., Terrillion C.E., Piantadosi S.C., Gould T.D. (2011). The Mouse Forced Swim Test. J. Vis. Exp..

[87] da Cruz J.F.O., Gomis-Gonzalez M., Maldonado R., Marsicano G., Ozaita A., Busquets-Garcia A. (2020). An Alternative Maze to Assess Novel Object Recognition in Mice. Bio Protoc..

[88] Bellot M., Espinosa-Velasco M., López-Arnau R., Escubedo E., Gómez-Canela C. (2022). Characterization of Monoaminergic Neurochemicals in Cortex and Striatum of Mouse Brain. J. Pharm. Biomed. Anal..

