# Supplementary material for: Acute Paraoxon-Induced Neurotoxicity in a Mouse Survival Model: Oxidative Stress, Dopaminergic System Alterations and Memory Deficits
Source: Int J Mol Sci. 2024 Nov 14;25(22):12248. doi: 10.3390/ijms252212248 (PMC11594717; doi:10.3390/ijms252212248)
Supplement: Supplementary file 1 [file ijms-25-12248-s001.zip › ijms-3293735-supplementary.pdf]

# SUPPLEMENTARY MATERIAL

## Acute Paraoxon-induced Neurotoxicity in a mouse survival model: Oxidative stress, Dopaminergic System Alterations and Memory Deficits

Edurne Urquizu[1], Selma Paratusic[1], Júlia Goyenechea[2], Cristian Gómez-Canela[2], Berta Fumàs[1], David Pubill[1], Demetrio Raldúa[3], Jordi Camarasa[1], Elena Escubedo[1], Raul López-Arnau[1*].

[1] *Department of Pharmacology, Toxicology and Therapeutic Chemistry, Pharmacology Section and Institute of Biomedicine (IBUB), Faculty of Pharmacy, University of Barcelona, 08028 Barcelona, Spain.*

[2] *Department of Analytical Chemistry and Applied (Chromatography Section), School of Engineering, Institut Químic de Sarrià – Universitat Ramon Llull, Barcelona, Spain.*

[3] *Institute for Environmental Assessment and Water Research (IDAEA-CSIC), 08034 Barcelona, Spain.*

*Corresponding author. email: raullopezarnau@ub.edu

## Racine Scale

Modified Racine Scale with minor modifications, adapted from Figueiredo et al., 2011; Racine, 1972.

- Score 0: Without behavioral response.
- Score 1: Behavioral arrest, orofacial movements, chewing.
- Score 2: Head nodding / myoclonus.
- Score 3: Forelimb clonus (unilateral/bilateral convulsion of upper limb), Straub tail reaction, extended position of the body.
- Score 4: Bilateral convulsion of extremities and rearing of torso.
- Score 5: Stereotypic movements and falls.
- Score 6: Complete tonic convulsions.

**Table S1.** Effects of the different paraoxon (POX) doses in the onset of S.E., the scores in the Racine Scale and the rate of survival after POX acute poisoning.

| POX dose | Onset of S.E. (min) | Racine Score (0-6) | Survival rate (%) |
|---|---|---|---|
| 2 mg/kg | 3-7 | 3-4 | 100% |
| 3 mg/kg | 3-5 | 4-5 | 100% |
| 4 mg/kg | 2-4 | 5-6 | 91.4% |

**Table S2.** Concentration of different neurotransmitters and metabolites expressed in pg/mg tissue ± SEM, in (A) HP, (B) PFC and (C) STR.

**(A)** **Hippocampus**

| | CTL | POX |
|---|---|---|
| | Concentration (pg/mg tissue) | Concentration (pg/mg tissue) |
| **Histam** | 13.75 ± 2.41 | 16.95 ± 5.21 |
| **ACh** | 1314.60 ± 53.71 | 1587.39 ± 105.30 |
| **Asp. Ac.** | 162122.58 ± 7762.44 | 169650.62 ± 10760.04 |
| **GABA** | 356482.45 ± 9249.30 | 327636.49 ± 23199.67 |
| **Tyr** | 14649.83 ± 1118.61 | 15126.30 ± 1172.79 |
| **L-DOPA** | 25.78 ± 4.21 | 33.15 ± 2.70 |
| **DA** | 13.71 ± 2.29 | 8.86 ± 0.68 |
| **DOPAC** | 1034.91 ± 122.65 | 791.34 ± 151.18 |
| **HVA** | 19499.43 ± 1473.00 | 18133.84 ± 1568.76 |
| **3-MT** | 178.87 ± 15.55 | 173.21 ± 53.05 |
| **NE** | 663.43 ± 23.46 | 612.55 ± 27.46 |
| **Trypt** | 4537.46 ± 272.00 | 4542.53 ± 535.82 |
| **Serot** | 488.58 ± 37.46 | 522.72 ± 61.04 |

**(B)**

**Prefrontal Cortex**

| | CTL | POX |
|---|---|---|
| | Concentration (pg/mg tissue) | Concentration (pg/mg tissue) |
| **Histam** | 17.69 ± 1.98 | 16.46 ± 1.05 |
| **ACh** | 1431.88 ± 115.90 | 1506.67 ± 124.35 |
| **Asp. Ac.** | 79563.57 ± 4175.49 | 79815.83 ± 6633.28 |
| **GABA** | 182613.91 ± 11178.44 | 174331.16 ± 2104.69 |
| **Tyr** | 18112.25 ± 1155.88 | 18211.49 ± 1371.05 |
| **L-DOPA** | 33.28 ± 2.56 | 31.30 ± 3.26 |
| **DA** | 153.26 ± 25.53 | 310.48 ± 47.43 |
| **DOPAC** | 2852.98 ± 493.68 | 3392.51 ± 402.13 |
| **HVA** | 16372.73 ± 862.03 | 15914.61 ± 1143.65 |
| **3-MT** | 395.42 ± 26.11 | 443.99 ± 52.20 |
| **NE** | 553.34 ± 46.16 | 599.28 ± 22.85 |
| **Trypt** | 4145.47 ± 302.73 | 3966.39 ± 335.57 |
| **Serot** | 571.23 ± 25.86 | 627.26 ± 51.01 |

**(C)**

**Striatum**

| | CTL | POX |
|---|---|---|
| | Concentration (pg/mg tissue) | Concentration (pg/mg tissue) |
| **Histam** | 18.47 ± 1.66 | 16.78 ± 1.27 |
| **ACh** | 1715.61 ± 138.55 | 1725.33 ± 147.63 |
| **Asp. Ac.** | 81916.25 ± 6843.04 | 76522.07 ± 6747.42 |
| **GABA** | 232230.25 ± 10971.86 | 246324.62 ± 13397.96 |
| **Tyr** | 14881.57 ± 850.00 | 137020.38 ± 1127.95 |
| **L-DOPA** | 46.68 ± 13.89 | 47.61 ± 6.19 |
| **DA** | 6974.82 ± 462.49 | 6866.95 ± 598.73 |
| **DOPAC** | 9804.14 ± 750.39 | 9781.34 ± 696.24 |
| **HVA** | 14303.57 ± 1070.57 | 11979.48 ± 1712.65 |
| **3-MT** | 9252.94 ± 550.71 | 9993.85 ± 836.50 |
| **NE** | 88.61 ± 11.23 | 98.67 ± 22.27 |
| **Trypt** | 4273.67 ± 352.59 | 4224.95 ± 480.93 |
| **Serot** | 608.38 ± 36.47 | 604.612 ± 62.19 |

**Figure S1.** Score and latency to fall obtained by control and POX treated mice in the Rotarod test. (A) The score obtained is presented as the maximum revolutions per minute reached by individuals in the selected session. (B) Latency to fall from is shown as the maximum time (in seconds) that subjects are able to stay in the rod. Data are expressed as mean ± SEM. (N=12-13/group).

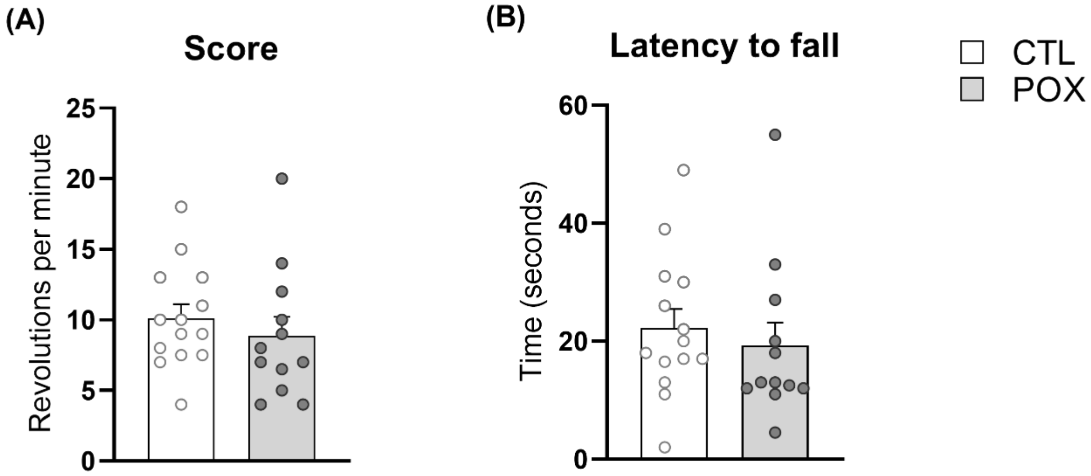


**Figure S2.** (A) Percentage of time spent in the closed and open arms of the EPM and (B) number of entries made in each of the arms by both the control and POX-treated groups. Bars represent mean ± SEM, two-way anova. N=12-14/group.

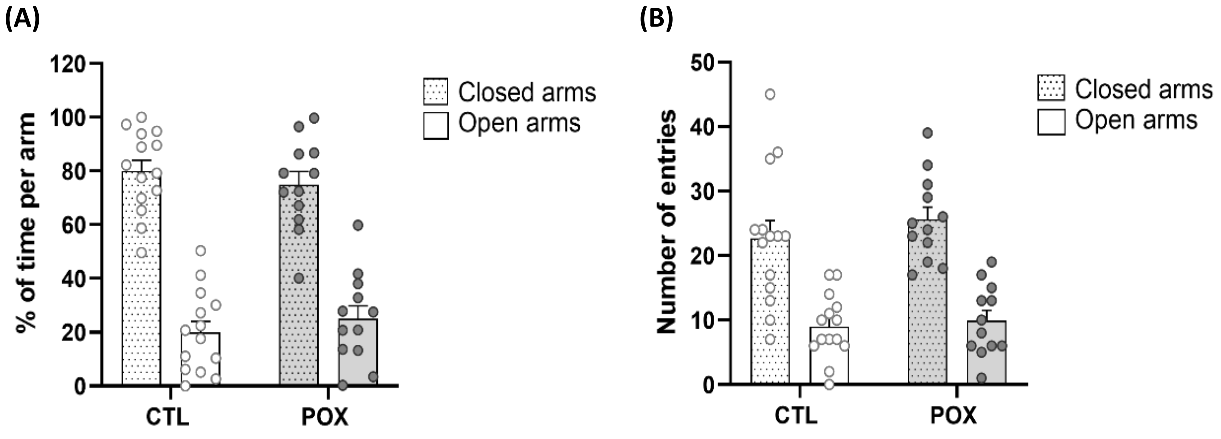

**Figure S3.** Immobility time (seconds) of control and POX-treated mice during FST. Data in bars are expressed as mean ± SEM. N=7/group.

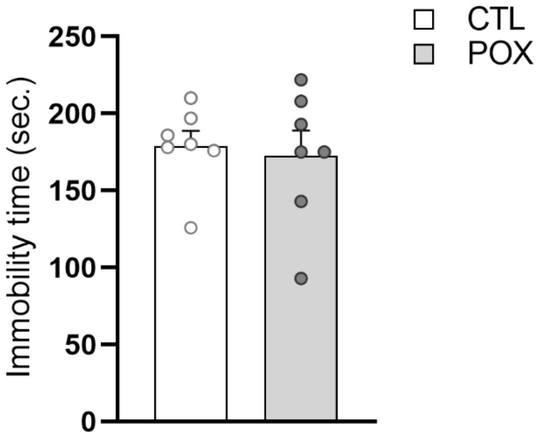


**Figure S4.** (A) Total exploration time during NOR test; (B) time exploring the familiar object; (C) time exploring the novel object, expressed in seconds. Data are expressed as mean ± SEM, *$p$<0.05. N=12/group.

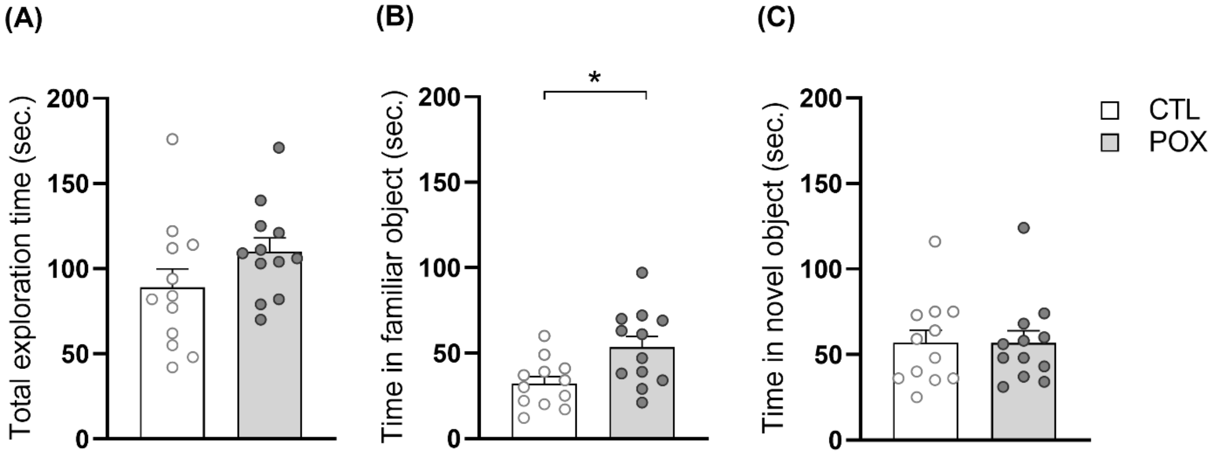

**Figure S5.** Neurotransmitter levels (expressed as percentage vs. corresponding control group) in STR. Bars represent mean ± SEM, *$p<0.05$, Student's t-test, N=3-7/group. Histam= histamine; Asp. Ac.= aspartic acid; GABA= gamma-aminobutyric acid; Tyr= tyrosine; L-DOPA= levodopa; DOPAC= 3,4-Dihydroxyphenylacetic acid; HVA= homovanillic acid; 3-MT= 3-Methoxytyramine; NE= norepinephrine; Tryp= tryptophan; Serot= serotonin.

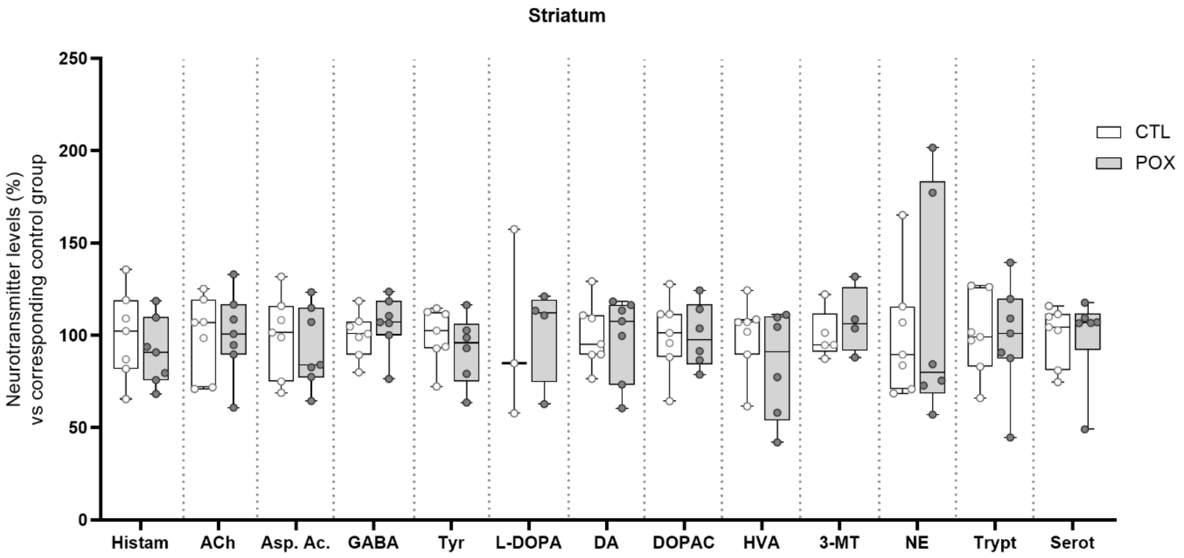

# Molecular Weight Markers

(A)

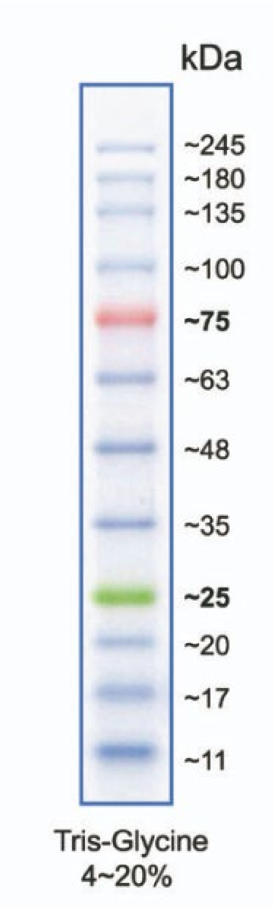


(B)

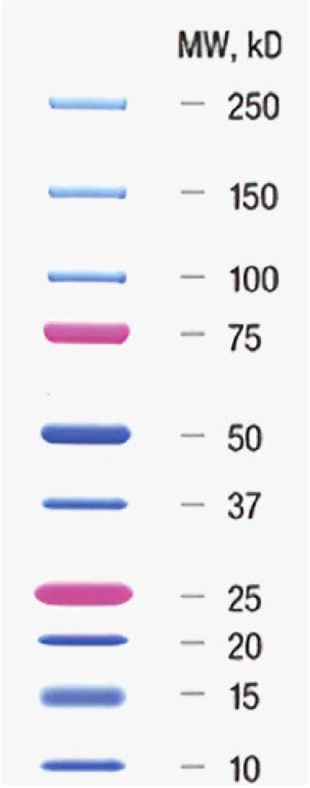


Molecular weight markers used to perform Western Blots. (A) BlueStar Plus Prestained Protein Marker (ref. MWP04; Nippon genetics, Germany). (B) Prestained Protein Standards (ref. 1610374; BioRad Laboratories, USA).

## REFERENCES


1. Figueiredo, T. H., Qashu, F., Apland, J. P., Aroniadou-Anderjaska, V., Souza, A. P., & Braga, M. F. M. (2011). The GluK1 (GluR5) Kainate/α-Amino-3-hydroxy-5-methyl-4-isoxazolepropionic Acid Receptor Antagonist LY293558 Reduces Soman-Induced Seizures and Neuropathology. *The Journal of Pharmacology and Experimental Therapeutics*, *336*, 303-312, https://doi.org/10.1124/JPET.110.171835
2. Racine, R. J. (1972). Modification of seizure activity by electrical stimulation: II. Motor seizure. *Electroencephalography and Clinical Neurophysiology*, *32*, 281-294, https://doi.org/10.1016/0013-4694(72)90177-0
